# Burden and Preparedness amongst Informal Caregivers of Adults with Moderate to Severe Traumatic Brain Injury

**DOI:** 10.3390/ijerph17176386

**Published:** 2020-09-02

**Authors:** Kirsten Lieshout, Joanne Oates, Anne Baker, Carolyn A. Unsworth, Ian D. Cameron, Julia Schmidt, Natasha A. Lannin

**Affiliations:** 1 Occupational Therapy, School of Allied Health, La Trobe University, Melbourne, Victoria 3086, Australia; kirsten.lieshout@gmail.com (K.L.); carolyn.unsworth@monash.edu (C.A.U.); julia.schmidt@ubc.ca (J.S.); 2 Kids Plus Foundation, Melbourne, Victoria 3216, Australia; 3 Evaluate, Sydney, NSW 2089, Australia; joanne@evaluate.com.au; 4 Occupational Therapy, School of Health, Federation University, Melbourne, Victoria 3000, Australia; a.baker@federation.edu.au; 5 Department of Neuroscience, Monash University, Melbourne, Victoria 3004, Australia; 6 Department of Rehabilitation, Jönköping University, SE-551 11 Jönköping, Sweden; 7 John Walsh Institute for Rehabilitation Research, Faculty of Medicine and Health, The University of Sydney, St Leonards, NSW 2065, Australia; ian.cameron@sydney.edu.au; 8 Department of Physical Therapy, University of British Columbia, Vancouver, BC V6T 1Z4, Canada; 9 Occupational Therapy Department, Alfred Health, Melbourne, Victoria 3004, Australia

**Keywords:** traumatic brain injury, caregivers, burden

## Abstract

This study examined the patterns of informal (unpaid) caregiving provided to people after moderate to severe traumatic brain injury (TBI), explore the self-reported burden and preparedness for the caregiving role, and identify factors predictive of caregiver burden and preparedness. A cross-sectional cohort design was used. Informal caregivers completed the Demand and Difficulty subscales of the Caregiving Burden Scale; and the Mutuality, Preparedness, and Global Strain subscales of the Family Care Inventory. Chi-square tests and logistic regression were used to examine the relationships between caregiver and care recipient variables and preparedness for caregiving. Twenty-nine informal caregivers who reported data on themselves and people with a moderate to severe TBI were recruited (referred to as a dyad). Most caregivers were female (*n* = 21, 72%), lived with the care recipient (*n* = 20, 69%), and reported high levels of burden on both scales. While most caregivers (*n* = 21, 72%) felt “pretty well” or “very well” prepared for caregiving, they were least prepared to get help or information from the health system, and to deal with the stress of caregiving. No significant relationships or predictors for caregiver burden or preparedness were identified. While caregivers reported the provision of care as both highly difficult and demanding, further research is required to better understand the reasons for the variability in caregiver experience, and ultimately how to best prepare caregivers for this long-term role.

## 1. Introduction

Following moderate to severe traumatic brain injury (TBI), many people experience significant and lifelong disability which results in them needing assistance from formal (paid) or informal (unpaid) caregivers to complete everyday activities that previously would have been completed independently [1]. Around two thirds of the care provided to people with severe TBI is provided by informal caregivers such as family members or friends [1,2,3]. Informal caregivers (herein referred to as caregivers) often report a considerable caregiver burden [4,5,6,7]. In this context, burden is a multidimensional concept which describes the ‘time demand’ and ‘felt difficulty’ associated with performing their caregiving role [4].

The considerable burden experienced by caregivers has been long acknowledged [8,9,10] and has been shown to increase with time [11,12]. In addition, the perceived burden for caregivers of adults with TBI has been suggested to be higher when compared to caregivers for adults with other conditions such as dementia [13]. For example, aggressive problems related to greater burden and poorer quality of life among caregivers for people with TBI compared to carers for people with dementia, and TBI carers experienced greater overall caring burden than carers of people with dementia. A systematic scoping review of caregiver outcomes and interventions reported in the TBI literature [7] has also shown that, while 62 papers discuss outcomes for caregivers, only 21 describe interventions to support caregivers. Similarly, Kreitzer, Kurowski and Bakas [14] also note that little research has been undertaken to investigate the interventions developed to support untrained caregivers. Therefore, while the burden of caregiving is well known, there is less understanding regarding the most effective ways to support caregivers in an effort to reduce their burden [11,14]. A first step to developing therapeutic interventions targeting caregiver burden and preparedness, is to better understand factors which influence burden levels.

Caregivers often report feelings of a lack of preparedness for the caregiving role [4,15,16,17,18,19], and report feeling obliged to assume this role because of gaps in service provision [20]. It has also been suggested that the sudden onset of TBI (compared to the slower onset of other neurological conditions) leaves the caregiver with little time to prepare and adjust to this new role [13]. The constructs of preparedness for caregiving and caregiver burden may be measured with caregivers at any time on their journey. Researchers using the Family Care Inventory, the Caregiving Burden Scale or interview have included participants who have had as little as six weeks, through to 20 years’ experience on their caregiving journey [16,21,22]. Therefore, a strength of this literature is that caregiver views are captured across their journey as some participants are more able to rate or discuss these issues immediately, while others are better placed to make comment after time, experience and reflection. Furthermore, a carer’s need for information, practical support and psychosocial resources changes over time as they adapt to their role and the person with TBI they care for adapts to living with functional limitations [23].

An understanding of the factors associated with caregiver burden for those caring for people after TBI might enable the development of effective interventions to prepare and support new caregivers in this role. A small number of studies have shown that predictors of caregiver burden include the level of overall disability and impaired executive functioning of the person with the TBI, and levels of assistance received [5,24]. However, no significant correlation between caregiver burden and the demographic data of the person with the TBI (e.g., gender, age) or injury severity (i.e., Glasgow Outcome Scale score) [5] has been shown in studies to date. Previous research has found a link between the concepts of caregiver burden and the preparedness for caregiving, suggesting that increasing a caregiver’s preparedness to provide caregiving is an important step in reducing longer-term caregiver burden [17].

Further research is thus needed to better understand the burden of caregivers for adults with moderate to severe TBI, to better prepare and support them in this role. The purpose of the current study was to (i) understand the patterns of informal caregiving for people after TBI, (ii) explore the relationship between caregivers/care needs and individuals with TBI, (iii) investigate factors that predict perceived caregiver burden and preparedness for caregiving.

## 2. Materials and Methods

A cross-sectional cohort study was conducted.

### 2.1. Participants

Caregivers of adults living with severe TBI in New South Wales (NSW, Australia) were recruited using both verbal invitation and advertisements. Caregivers were eligible for inclusion in this study if they were providing informal care at the time of the study for an adult who had sustained a moderate to severe TBI. For this study, we included participants who cared for adults with a post-traumatic amnesia (PTA) level using the Westmead PTA Scale [25] of >7 days. We used the Jennett and Teasdale [26] classification of <5 min very mild, 5–60 min mild, 1–24 h moderate, 1–7 days severe, 1–4 weeks very severe and >4 weeks extremely severe. In addition to this score, participants also needed to have a Glasgow Coma Scale (GCS) [27] of 3 or more, or a Care and Needs Scale (CANS) of ≥4. For the Glasgow Coma Scale (GCS), we used the classification of minor (score of 15), mild (13–14), moderate (9–12) and severe (3–8) [28]. For the carer-reported CANS, we determined that a score of ≥4 on the Care and Needs Scale (CANS) [29] at the time of hospital discharge (although retrospectively calculated) indicated that the person with the TBI required nursing care, assistance or supervision for basic activities of daily living, and/or supervision at the time of discharge from hospital care, which equated to having significant care needs. Given the complexity of combining the findings of these different scales, we reasoned that we could best describe our sample as having a moderate to severe head injury.

All patients with a TBI had sustained their injury ≥12 months ago and lived in private housing or a community group home in NSW, Australia. The research team included participants with both long-term and recent experiences in their caregiving journey, in order to be inclusive of a variety of experiences and perspectives. Therefore, it was anticipated that caregivers would have a wide variety of experience in this role, and that the results would need to acknowledge differences in length of time providing care. Caregivers for children and/or young people (<18 years) with a TBI were excluded, as were caregivers whose primary language was not English, as funding was not available for interpreter services.

### 2.2. Instruments

Demographic and injury data, as well as functional ability information, were collected on all participants. Clinical measures of the functional ability, care needs and caregiver demands were also completed, including the Functional Independence Measure (FIM™) [30], the CANS [29], Family Care Inventory [15] and the Caregiving Burden Scale [31]. The FIM was administered to determine the level of physical and cognitive independence in everyday tasks. The FIM has excellent inter-rater reliability [32], excellent test-retest reliability and excellent internal consistency [33]. The Family Care Inventory [15,21,31] measures the perceived burden and preparedness of caregivers. Qualitative and quantitative responses were captured through the use of all subscales of the Family Care Inventory, including the Burden, Preparedness for Caregiving and Global Strain subscales. The burden subscale measures across three dimensions: objective demand (which is defined as perceived disruption of the caregiver’s life), subjective demand (which is defined as the extent to which the caregiver perceives care responsibilities to be overly demanding), and subjective stress (which is defined as the emotional impact of caregiving). The reliability of these questionnaires has been shown to range from satisfactory to excellent when used with informal caregivers [34]. The Caregiving Burden Scale [31] measures the perceived burden and preparedness of caregivers, capturing both qualitative and quantitative responses. Caregivers were asked to rate whether they provided support to provide the tasks, and then rated how difficult it is (Demand Difficulty subscale) on a Likert-scale, from 0 (none) to 4 (a great deal). The CANS measures the type and extent of support required by people with a TBI [29]. Both sections of this scale were used in this study: (1) the Needs Checklist, which outlines the type and range of support required, and (2) the Support Levels, which outline the extent of support as rated on eight levels [29,35,36].

### 2.3. Procedure

Informed written consent to participate in the study was obtained. Experienced occupational therapists visited caregivers in their own residence to gather demographic data and provide each caregiver with an opportunity to self-complete the questionnaires.

### 2.4. Ethical Standards

Institutional and ethical approval was received from La Trobe University and Royal Rehabilitation Centre Sydney Human Research Ethics Committees (ethical approval number 404/12). The authors assert that all procedures contributing to this work comply with the ethical standards of the relevant national and institutional committees on human experimentation and with the Helsinki Declaration of 1975, as revised in 2008.

### 2.5. Data Analysis

Data were analyzed using the Statistical Package for Social Sciences Version 26.0 (IBM Corp: Armonk, NY, USA) [37]. Datasets with missing data were included in analyses, with missing data coded as such. As participants’ time since injury varied greatly, the group was divided into two sub-groups (i.e., 1–3 years post-injury and >3 years post-injury), as this created two groups of sufficient size to support meaningful description of participants who were still new to caregiving, or who had been caregiving for a comparatively longer time. Descriptive statistics were used to summaries demographic data. Chi square tests were used to examine the relationship between the demographic data of caregivers and independent variables (such as care needs measured on CANS and level of physical and cognitive disability on the FIM). Strengths of association were determined according to the guidelines specified by Portney and Watkins [38]. Non-parametric tests were used to ascertain the differences between two population groups within the sample on a single, ordinal variable, without specific distribution. Logistic regression was used to explain and predict caregiver burden and preparedness, and the relative contribution of caregiver gender, relationship quality (as measured by the Mutuality subscales of the Family Care Inventory), and level of care needs (measured using the Caregiving Burden Scale). Variables significantly associated with caregiver burden were entered into the model as independent variables. Associations were considered as statistically significant if *p* < 0.05. Regression analyses were performed with data from the total sample, and also data from the two subgroups (by time post injury).

## 3. Results

### 3.1. Demographic Information Concerning Dyads

Twenty-nine informal caregivers reporting information for themselves and a person with a moderate to severe TBI (described as a dyad), participated in the study at a mean of 7.8 (SD 7.9) years post-injury (range 1–28 years). Twelve of the people with a TBI had been injured 1–3 years ago and 17 had sustained their TBI for more than three years (see Table 1). Most informal caregivers were female (*n* = 21, 72%), lived with the person with TBI (*n* = 20, 69%), and were most frequently the mother of the person with the TBI (*n* = 12, 41%) (see Table 2). Persons with the TBI had a mean age of 42 (*SD* 15.3) years, and 13 (45%) were classified as having very high care needs (CANS Level 7, meaning they could not be left alone for any period of the day or night). Care needs were most frequently met solely by the informal caregivers (*n* = 13, 45%), although some were supported by a combination of both formal and informal caregivers (*n* = 9, 31%). An outlier dyad was noted in our sample. One caregiver provided support for a person who scored a Level 1 on the CANS at the time of hospital discharge (indicating low care needs). However, this participant still met our inclusion criteria based on Westmead PTA score and GCS.

### 3.2. Patterns of Informal Caregiving Following TBI

**Figure 1:**
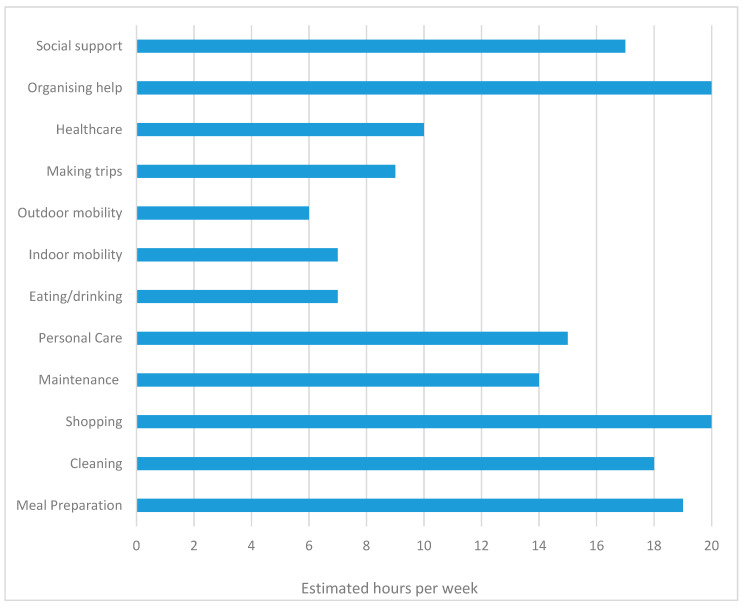
Self-reported estimated hours of care per week attributed to caregiving tasks.

Informal caregiving activities were reported through the CANS (Figure 1). Caregiving activities were widespread and took a large amount of time. For example, caregivers reported spending time organizing help, aids or adaptations (20 h/week), shopping (20 h/week), preparing meals (19 h/week), cleaning (18 h/week), providing social support (17 h/week), and providing personal care (15 h/week).

The mean level of caregiving burden/demand on the subscale of the Caregiving Burden Scale for the whole sample was rated as 0.62 (*SD* 0.19, range 0.25–0.93), suggesting a high level of caregiving demand, and caregivers rated the level of difficulty of undertaking caregiving activities at a mean of 1.81 (*SD* 0.41, range 1.04–2.74), indicating a high level of difficulty. On the Family Care Inventory, objective burden was rated higher by the whole group (mean 13.9, or 50% of total score), in comparison to subjective burden (mean 6.6, 33% of total score) or subjective demand (mean 3.5, or 18% of total score); higher scores suggest higher levels of perceived burden. Further, some caregiving activities on the Caregiving Burden Scale were rated by caregivers as both highly demanding and difficult. Service coordination tasks (e.g., arranging doctor’s appointments, making healthcare decisions, organizing services and informing the doctor or family about the health of the person with the TBI) had a high demand score of 0.77 (*SD* 0.14, range 0.43–0.93) and a high difficulty score of 1.83 (*SD* 0.67, range 1.04–3.67), suggesting that this activity contributes greatly to perceived burden.

### 3.3. Caregiver Burden and Preparedness for Caregiving

Most caregivers (*n* = 21, 72%) reported that they felt “well prepared” or “very well prepared” overall for caregiving on the preparedness for caregiving subscale of the Family Care Inventory. For the whole sample, caregivers felt most prepared for taking care of the individuals’ physical needs (*n* = 21, 72%) and responding to the individual’s needs in an emergency (*n* = 18, 62%). In contrast, caregivers reported that they felt the least prepared to get help and information from the health system (*n* = 6, 20%) and to deal with the stress of caregiving (*n* = 6, 21%). The most frequent areas of concern for those caregivers who responded to this subscale were the individual’s health condition (Quite a bit, or A Great deal, *n* = 17, 86%), and who would provide care for the individual after the caregiver dies (Quite a bit, or A Great deal, *n* = 16, 63%) (Table 3). Just over half the caregivers reported that they had decreased time for themselves (*n* = 13, 54%) and experienced added tension in life (*n* = 13, 54%). Despite this, caregivers reported experiencing very little relationship stress (either “none” or only a “small amount”, *n* = 15, 63%).

### 3.4. Relationships between Caregiver Demographic Data and Caregiver Burden and Preparedness

The caregiver demographic variables, including relationship to the person with the TBI (*p* = 0.697), cohabitation with the person with the TBI (*p* = 0.107), level of care needs on CANS (*p* = 0.792), level of cognitive and physical disability on FIM™ (*p* = 0.741), body mass index of person with the TBI (*p* = 0.391), and number of helpers and number of services received (*p =* 0.422) were not significantly related to the variables of caregiver burden or preparedness (*p* > 0.05 for all comparisons).

### 3.5. Predicting Perceived Preparedness for Caregiving and Caregiver Burden

A series of regression analyses were performed with data from the total sample, and data from the two subgroups (divided by time post injury), but none were found to be statistically significant. This included whether any of the demographic data (caregiver gender, relationship to the person with the TBI, cohabitation with the person with the TBI, level of cognitive and physical disability (FIM), length of inpatient stay and CANS level of care needs) were able to predict the caregiver preparedness rating score. Similarly, no predictors of caregiver burden were found when considering the person with the TBI’s level of cognitive and physical disability (FIM™), geographical location, time since injury (between 1–3 years), CANS level of care needs, total number of services and help received. Regression analyses did not identify any significant findings when examining if caregiver gender, relationship to the person with the TBI, cohabitation, time since injury, total number of services and help received predicted caregiver difficulty (burden).

## 4. Discussion

Consistent with previous studies, this study found that caregivers had a high level of demand and difficulty in their role [8,9]. The patterns of informal caregiving highlighted in this study demonstrated that people with a moderate to severe TBI are supported by both family and paid support, noting that 12 of the 29 people with TBI had some financial assistance through no-fault insurance or compensation systems. None of our sample of people with a moderate to severe TBI were able to live alone without support post-discharge, and the majority re-located to live in the care of their family. Regardless of time post-injury, caregivers reported the demanding nature of balancing the support required for everyday essential tasks for the person (e.g., self-care) and the difficulty of coordinating services for the person with the TBI. In considering responses across all scales, many informal caregivers reported that overall, they were relatively well prepared for taking on their caregiving role, but caregivers of adults in both groups (injured 1–3 years ago, or >3 years) continued to report a high level of concern about the future care needs of their family member. Consistent with other literature, a strength of this study was the range of time that participants had been caregivers, thus providing reflections and ratings of burden and preparedness for caregiving at different time points on the caregiving journey [16,21,22].

The responsibility of providing care post-discharge for people with a moderate to severe TBI in Australia is usually assumed by informal family members, hypothesized to be a necessity due to the limited availability of adequate formal services available to people when living in community settings (i.e., outside a residential care setting) [3,10]. Furthermore, mothers of adults with TBI were the most frequent caregivers in our sample, and therefore, it is understandable that they expressed concern about who was going to provide care when they are no longer able. The limited accommodation options available for people with severe TBI [10], inappropriateness of placement into aged care facilities for younger people with a severe TBI [39,40], and the difficult, lengthy, and financially expensive process of sourcing these options [41] means that informal caregivers are thrust into their role, whether they feel prepared to provide this care or not. Within our study, even those caregivers who felt prepared for their role continued to express a level of concern about the future and who would care for the person living with the TBI if they were unable to care.

In our sample, there were mixed levels of preparedness reported by the informal caregivers, which is in keeping with previous research on informal (unpaid) caregivers [4,16,18,19,22,34]. Although a chi square analysis was not able to be performed due to low sample size in cells, there was a trend that the caregivers who reported feeling “very well prepared” for caregiving; mostly from the group who were caring for a person who had received their TBI more than 3 years ago. This suggests that caregivers may develop a sense of preparedness over time [13,17]. It is also possible that caregivers take time to arrive at a point of perceiving some of the rewards from caregiving [17], and further research is required to better support caregivers to both psychologically reframe the burden, and physically manage the load [42]. In fact, as Baker et al. [7] comment “…while literature on the negative outcomes of caregiving is close to reaching data saturation, positive caregiving out-comes remain scarcely studied.” (p. 58). Therefore, further funding and research needs to be invested, to ensure positive outcomes for caregivers. This may include strengthening formal and informal support networks, providing ongoing education or skill building, for example in the areas of problem solving and accessing existing services. Studies on interventions that primarily support the caregiver have been found to be more effective than those targeting caregiver and survivor dyads [14].

We acknowledge that the small sample size limits the generalizability of our results and the predictive validity of study variables. Additionally, we note that classification of severity of head injury varies across this literature, making comparisons between studies challenging. However, the care needs of all persons with head injuries in our study were considerable, despite differing scores across the severity measures we used (Westmead PTA scale and GCS), and similar to the broader population of individuals needing caregiver support after TBI [11]. We also note that the CANS scores were based on retrospective accounts of care from the caregivers, and further research is needed to understand any bias in such reports, as this tool was developed for use by clinicians [29]. As such, we acknowledge that a ‘sense-of-duty’ or feelings of responsibility may have influenced how caregivers responded to items about burden or difficulty. We also note that only 13 participants had CANS scores, thus reducing opportunities to find relationships with other variables, if they exist. Our study also found carers estimated high care time for the person with TBI. However, it is a known limitation that caregivers overestimate the time spent, as time is inherently difficult to reliably quantify [43,44]. Future studies should investigate methods of objectively measuring care times. Finally, caregiver burden relates to perceived stress, but not always. Further studies that tease out these concepts, and how to alleviate perceived stress, are also required.

## 5. Conclusions

In our study, no variables were found to significantly predict caregiver burden nor caregiver preparedness. This finding is not in keeping with the majority of previous literature [5,6,17], where a person’s participation restrictions and impaired executive functioning have previously predicted levels of caregiver burden. Reasons for this discrepancy are not clear, with our sample demographics and size being similar to these prior studies [5,24]. Most likely, the lack of significant predictors for caregiver burden and preparedness reflects the complex nature of these constructs in the more severe TBI population. Clearly, clinicians should consider each caregiver–person with a TBI dyad as unique and tailor preparation and ongoing support to meet individual needs. Further research should also consider triangulating the data collection, by conducting in-depth interviews with caregivers to gain additional insights into the complexities of the constructs of burden and preparedness. There is limited knowledge of the role of informal caregivers supporting adults to live in the community after moderate to severe TBI, despite the reliance in Australia on these informal caregiver networks. In particular, more research is required on strategies and education programs to support caregivers to identify and promote the rewards associated with caregiving. These findings demonstrate the importance of continued research in this area, to ascertain how informal caregivers can be better supported in assuming and maintaining their caregiving role. Research investigating the most effective strategies to provide adequate preparation, training and support to informal caregivers of people with a moderate to severe TBI is still required, to ensure that each person with TBI is transitioned to community living with the greatest amount of support.

## Figures and Tables

**Table 1:** Demographic characteristics of informal caregivers of people with a traumatic brain injury.

Carer Characteristics	Sample (*n* = 29, %)	Injured 1–3 Years Ago (*n* = 12, %)	Injured >3 Years (*n* = 17, %)
Gender, female, *n* (%)	21 (72)	9 (75)	12 (71)
Time known person with TBI, years, mean (*SD*)	32.0 (11)	26.6 (11)	35.8 (10)
Currently a live-in caregiver, *n* (%)	20 (69)	10 (83)	10 (59)
Time lived with person with TBI, years, mean (*SD*)	17.5 (13)	19.7 (15)	15.8 (12)
Relationship to person with traumatic brain injury, *n* (%)			
Wife/female partner	4 (14)	3 (25)	1 (6)
Husband/male partner	3 (10)	1 (8)	2 (12)
Son	1 (3)	0 (0)	1 (6)
Mother	12 (41)	4 (33)	8 (47)
Father	2 (7)	1 (8)	1 (6)
Other relative	6 (21)	2 (17)	4 (24)
Neighbour/friend	1 (3)	1 (8)	0 (0)
Change in caregiver working status, *n* (%)			
No change to work	3 (11)	2 (17)	1 (6)
Reduced hours, but still working	3 (11)	2 (17)	1 (6)
Unable to work	5 (18)	2 (17)	3 (19)
Not applicable/not working at time of injury	18 (62)	6 (50)	12 (71)

TBI: traumatic brain injury.

**Table 2:** Demographic characteristics of people with a traumatic brain injury (TBI).

Characteristics of People with TBI	Sample (*n* = 29, %)	Injured 1–3 Years Ago (*n* = 12, %)	Injured >3 Years (*n* = 17, %)
Gender, male, *n* (%)	22 (76)	10 (83)	12 (71)
Geographical location, *n* (regional, %)	11 (38)	3 (25)	8 (47)
Pre-injury education, *n* (%)			
University (under- and post-graduate)	4 (48)	6 (50)	8 (47)
Highschool completion	9 (31)	3 (33)	6 (36)
Less than 10 years of schooling	5 (17)	2 (17)	3 (18)
Missing	1 (3)	1 (8)	0 (0)
Cause of traumatic brain injury, *n* (%)			
Road traffic accident	16 (55)	5 (42)	11 (65)
Fall	10 (35)	7 (58)	3 (18)
Assault	2 (7)	0 (0)	2 (12)
Other	1 (3)	0 (0)	1 (6)
Age at time of injury, year, mean (*SD*)	42.4 (15.3)	42.5 (19.2)	42.3 (12.5)
Time post-injury, years, mean (*SD*)	7.8 (7.9)	2.1 (0.9)	11.3 (8.3)
Glasgow Coma Scale (GCS), mean (*SD*), range	6.6 (3.7), 3–13	7 (4), 3–13	6 (3), 3–11
Westmead Post-Traumatic Amnesia score			
Very severe TBI (>7 days) *n* (%)	29 (100)	12 (100)	17 (100)
Caregiving Burden Scale (Demand Difficulty subscale, 0–4), mean (*SD*)	1.44 (0.80)	1.3 (0.92)	1.52 (0.71)
Care and Needs Scale (CANS) total, *n* (%)			
Cannot be left alone (Level 7)	13 (45)	4 (33)	9 (53)
Can be left alone for a few hours (Level 6)	8 (28)	4 (33)	4 (24)
Can be left alone for part of the day, but not overnight (Level 5)	5 (17)	2 (17)	3 (18)
Can be left alone for part of the day and overnight (Level 4)	2 (7)	1 (8)	1 (6)
Can be left alone, but needs intermittent support (Level 1)	1 (3)	1 (8)	0 (0)
Functional Independence Measure: physical subscale, mean (*SD*); total score 91	55.9 (28.7)	56.9 (29.4)	55.3 (29.2)
Functional Independence Measure: Cognitive subscale, mean (*SD*); total score 35	20.0 (8.5)	21.5 (8.1)	19.0 (8.9)
History of pressure sores, yes, *n* (%)	8 (28)	3 (33)	5 (39)
Missing	7 (24)	3 (33)	4 (24)
Body mass index, obese, *n* (%)	8 (28)	2 (17)	6 (36)
Discharge destination, *n* (%)			
Home	22 (76)	10 (83)	12 (71)
Group home	2 (7)	1 (8)	1 (6)
Nursing home	2 (7)	1 (8)	1 (6)
Other	2 (7)	0 (0)	2 (12)
Missing	1 (3)	0 (0)	1 (6)
Main reason for accommodation change post-injury (*n* = 18), *n* (%)			
Care needs	13 (72)	5 (83)	8 (67)
Physical needs	3 (17)	1 (17)	2 (17)
Other reason	2 (11)	0 (0)	2 (17)
Living arrangements, *n* (%)			
With family, informal support only	13 (45)	7 (58)	6 (35)
With family, paid and informal support	7 (24)	3 (25)	4 (24)
Alone, no support	0 (0)	0 (0)	0 (0)
Alone, paid support only	3 (10)	1 (8)	2 (12)
Alone, paid and informal support	2 (7)	0 (0)	2 (12)
Group home	4 (14)	1 (8)	3 (18)
Compensatory status, *n* (%)			
No compensation	16 (55)	7 (58)	9 (53)
Insurance/Compensation scheme	8 (28)	3 (25)	5 (29)
Workplace injury	5 (17)	2 (17)	3 (16)
Working/studying, *n* (%)			
Pre-injury	28 (97)	11 (92)	17 (100)
Post-injury	3 (10)	0 (0)	3 (18)

TBI: traumatic brain injury; Care and Needs Scale: CANS; Glasgow Coma Scale: GCS.

**Table 3:** Areas of concern reported by informal caregivers, as indicated by the Family Care Inventory (*n* = 29).

Family Care Inventory Items	Not at All	A Little	Some	Quite a Bit	A Great Deal	Missing
Health condition of person with TBI	1 (3)	2 (7)	7 (24)	7 (24)	10 (35)	2 (7)
Obtaining help	0 (0)	7 (24)	7 (24)	7 (24)	6 (21)	2 (7)
Mood of person with TBI	3 (10)	4 (14)	9 (31)	7 (24)	4 (14)	2 (7)
Financial problems	3 (10)	5 (17)	9 (31)	2 (7)	8 (28)	2 (7)
Caregivers ability to continue care, if health of person with TBI worsened	4 (14)	6 (21)	6 (21)	7 (24)	3 (10)	3 (10)
Caregivers ability to continue care, if their health worsened	7 (24)	4 (14)	5 (17)	4 (14)	5 (17)	4 (14)
Having to leave the person with TBI alone	3 (10)	4 (14)	5 (17)	3 (10)	11 (38)	3 (10)
Whether professional care and advice received is adequate	5 (17)	7 (24)	4 (14)	6 (21)	4 (14)	3 (10)
Who will provide care if something happens to the caregiver	2 (7)	4 (14)	5 (17)	6 (21)	10 (34)	2 (7)
Concerned about the Future	1 (3)	9 (31)	6 (21)	3 (10)	7 (24)	3 (10)
Negative effect on the rest of the family	9 (31)	5 (17)	6 (21)	1 (3)	5 (17)	3 (10)
Nursing home placement	12 (41)	4 (14)	3 (10)	0 (0)	7 (24)	3 (10)
Equipment safety	18 (62)	2 (7)	1 (3)	2 (7)	1 (3)	5 (17)

TBI: traumatic brain injury.
